# Microbiome and Metabolome Illustrate the Correlations Between Endophytes and Flavor Metabolites in *Passiflora ligularis* Fruit Juice

**DOI:** 10.3390/ijms26052151

**Published:** 2025-02-27

**Authors:** Meijun Qi, Xuedong Shi, Wenlong Huang, Qilong Wei, Zhenwei Zhang, Rongqi Zhang, Shilang Dong, Sumera Anwar, Hafiz Faiq Bakhat, Butian Wang, Yu Ge

**Affiliations:** 1 College of Tropical Crops, Yunnan Agricultural University, Pu’er 665099, China; qimeijun2003@163.com (M.Q.); shixuedong2023@163.com (X.S.); huangwenlong23@163.com (W.H.); weiqilong20000@163.com (Q.W.); zhangzhenwei202309@163.com (Z.Z.); zhangrongqi07@163.com (R.Z.); dongshilang2003513@163.com (S.D.); 2 Department of Botany, Government College Women University Faisalabad, Faisalabad 38000, Pakistan; anwer_sumera@yahoo.com; 3 Department of Environmental Sciences, COMSATS University Islamabad, Vehari-Campus, Vehari 61100, Pakistan; faigsiddique@ciitvehari.edu.pk

**Keywords:** sweet granadilla, flavor biosynthesis, endophytic microbiota, volatile organic compounds, metabolomic profiling

## Abstract

This study investigates the interplay between volatile and non-volatile flavor metabolites and endophytic microbial communities during three developmental stages of *Passiflora ligularis* fruit juice. Using bioinformatics and metabolomics, we characterize microbial diversity and metabolic variations to understand flavor development. A total of 1490 bacterial and 1158 fungal operational taxonomic units (OTUs) were identified. Young fruits had higher microbial diversity, dominated by Proteobacteria and Firmicutes (bacteria) and Ascomycota and Basidiomycota (fungi). As the fruit matured, Proteobacteria increased while Firmicutes decreased, indicating that microbial succession is tied to development. Metabolomic profiling identified 87 volatile and 1002 non-volatile metabolites, with distinct chemical classes varying across stages. Saturated hydrocarbons and fatty alcohols were the main volatile metabolites, while organic acids and lipids among non-volatile metabolites showed stage-dependent changes, influencing flavor complexity. Correlation analysis showed microbial-flavor interactions: Proteobacteria negatively correlated with metabolites, while Firmicutes positively correlated with metabolites. Ascomycota positively correlated with volatile metabolites, whereas Basidiomycota showed an inverse relationship, highlighting their differential contributions to flavor biosynthesis. This study enhances understanding of microbial and metabolic factors shaping *P. ligularis* fruit flavor, highlighting the importance of microbial influence on fruit metabolomics. The findings suggest the potential for microbiome engineering to improve flavor quality, aiding postharvest management and industrial processing in the food and beverage industry.

## 1. Introduction

*Passiflora ligularis* Juss., known as sweet granadilla, belongs to the genus *Passiflora* within the Passifloraceae family alongside *P. edulis* Sims (passion fruit) [1]. The suitable climate for growth is in cool subtropical areas with temperatures between 15 and 18 °C, annual precipitation between 600 and 1000 mm, and elevation between 900 and 2700 m [1]. Peru is the primary producer, but its cultivation has expanded to other regions, including China, where it is grown in suitable subtropical areas such as Yunnan, Guangdong, Fujian, and Guangxi provinces [2]. The fruit has a smooth, orange-yellow rind, an oval shape, and a translucent aromatic pulp. Noted for its distinctive flavor with lychee, banana, and guava-like notes, *P. ligularis* is rich in amino acids, vitamin C, and vitamin E, making it valuable for fresh consumption and juice production. Due to its unique sensory attributes and nutritional benefits, it has gained attention for industrial processing [1].

The flavor of plant-based foods is primarily determined by a complex interplay of metabolites, including sugars, organic acids, amino acids, and volatile compounds [3,4]. Sugars (e.g., sucrose, glucose, fructose) and organic acids (e.g., citric acid, malic acid) influence sweetness and fruitiness through glycolysis and the tricarboxylic acid cycle [5,6]. Amino acids contribute to umami, sweet, bitter, and aromatic flavors [7]. Volatile compounds, synthesized via metabolic pathways such as the lipoxygenase, mevalonate, and shikimate pathways, produce key aroma components, including esters, terpenoids, and phenylpropanoids [8,9,10]. These flavor-related metabolites accumulate during fruit ripening, enhancing the sensory appeal of various *Passiflora* species [11,12]. However, the metabolic changes responsible for flavor variations in *P. ligularis* remain unexplored. Understanding these mechanisms is crucial for optimizing flavor quality in postharvest management and industrial applications.

Recent studies suggest that microbial communities play a crucial role in modulating fruit flavor through metabolic interactions. The plant microbiome comprises diverse microorganisms, including epiphytes and endophytes, that influence plant health and development [13,14]. Endophytes, colonizing plant tissues without causing diseases, include bacterial taxa such as Proteobacteria, Firmicutes, and Actinobacteria, as well as fungal groups like Ascomycota and Basidiomycota [15,16,17,18,19,20]. These microbial communities are shaped by plant genotype, age, physiological state, and environmental factors such as altitude and humidity [20,21,22,23,24,25,26].

Studies have shown that endophytes influence fruit quality by modulating metabolic pathways involved in nutrient absorption and secondary metabolite biosynthesis [27]. Endophytes facilitate the uptake of essential nutrients (C, N, P, K, Ca, Mo) and produce plant hormones like gibberellins (GA) and indole acetic acid (IAA), enhancing fruit development and quality [28,29]. Certain bacterial genera, such as *Bacillus*, contribute to plant growth by synthesizing aromatic compounds, polysaccharides, and lipids [29,30]. Additionally, microbial interactions within the endosphere create metabolic synergies that amplify signals influencing plant physiology [31,32]. Endophytes have demonstrated potential as biofertilizers, biostimulants, and biocontrol agents in sustainable agriculture [33,34,35,36].

Given the significance of endophyte-mediated metabolic processes, studying their role in *P. ligularis* fruit flavor development is of great importance. While previous research has linked endophytic microbial communities to plant health and growth [37], their influence on fruit flavor remains unclear. This study aims to elucidate the relationship between endophytic microbiota and metabolite profiles in *P. ligularis* fruit juice across different developmental stages. A comprehensive microbes–metabolites analysis was conducted to assess the correlation between microbial community dynamics and flavor compound accumulation, providing insights into potential microbiome-based strategies for flavor enhancement in industrial processing and postharvest management.

## 2. Results

### 2.1. Endophytic Microbial Communities in the Developing Passiflora ligularis Fruit Juice

**Figure 1:**
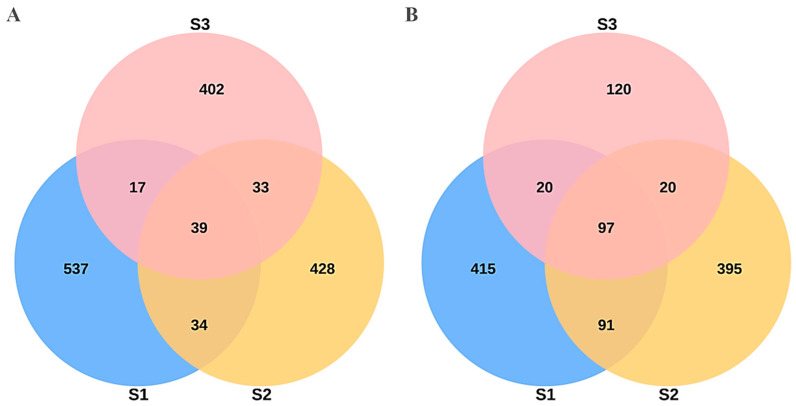
Venn diagrams illustrating the distribution of (**A**) bacterial and (**B**) fungal OTUs in the fruit juices of three developmental stages of *P. ligularis*. S1, S2, and S3 represent the young fruit stage (0–45 days after fruit setting), coloration stage (46–59 days after fruit setting), and maturity stage (60–79 days after fruit setting), respectively. The number within each region denotes the OUT counts, while the circle’s areas are not drawn to scale.

Microbial diversity analysis revealed dynamic changes in bacterial and fungal communities across fruit developmental stages. A total of 1490 bacterial OTUs and 1158 fungal OTUs were identified, with microbial richness decreasing as the fruit matured. Venn diagrams (Figure 1A,B) illustrate the number of shared and unique OTUs among the three developmental stages. For bacterial OTUs (Figure 1A), a total of 39 OTUs were shared across all three stages, while each stage contained distinct OTUs, indicating microbial succession. The highest number of unique bacterial OTUs was observed in S1 (537 OTUs), suggesting a richer bacterial community in the early stages of fruit development, while S3 exhibited a reduced diversity (402 unique OTUs).

Similarly, fungal OTU distribution (Figure 1B) revealed 97 core OTUs shared among all three stages, with 120 unique fungal OTUs in S3, reflecting a shift in the fungal community structure during fruit maturation. The fungal OTU richness was highest in S1 (415 unique OTUs) and gradually declined across S2 and S3. These results indicate that both bacterial and fungal communities undergo compositional shifts as the fruit develops, potentially influencing metabolite production and fruit flavor. Statistical summaries of OTU sequencing quality are provided in Supplementary Tables S1 (bacteria) and S2 (fungi).

#### 2.1.1. Bacterial Community Composition and Structure

**Figure 2:**
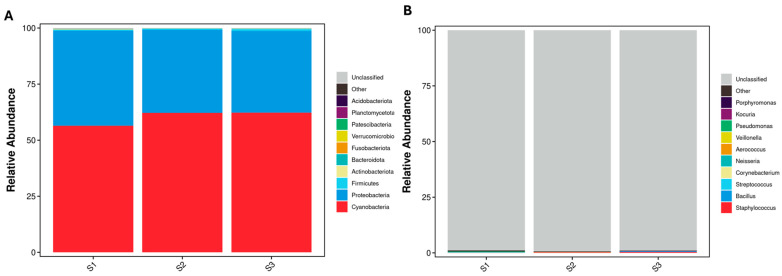
Relative abundance of bacterial communities in the fruit juices of three developmental stages of *P. ligularis*. (**A**) Stacked bar chart displaying bacterial phyla across S1, S2, and S3. (**B**) Stacked bar chart showing bacterial genus-level composition across S1, S2, and S3. The analysis was based on 16S rRNA sequencing. S1, S2, and S3 represent the young fruit stage (0–45 days after fruit setting), coloration stage (46–59 days after fruit setting), and maturity stage (60–79 days after fruit setting), respectively.

The 16S rRNA V3–V4 sequencing identified five major phyla: Proteobacteria (60.31%), Firmicutes (38.58%), Actinobacteriota (0.61%), Bacteroidota (0.14%), and Fusobacteriota (0.08%) in *P. ligularis* fruit juice. Proteobacteria and Cyanobacteria are the most abundant groups, while Firmicutes, Actinobacteriota, and Bacteroidota were present in lower proportions (Figure 2A). Proteobacteria abundance decreased, whereas Cyanobacteria increased from S1 to S3, indicating microbial succession during fruit maturation. Statistical comparison revealed a significantly higher abundance of Proteobacteria, Firmicutes, and Bacteroidota in S1 compared to S2 (Table S3).

At the genus level, 211 bacterial genera were detected, though unclassified bacteria made up the dominant group (>98.89%) (Figure 2B). Among classified genera, *Neisseria*, *Bacillus*, *Streptococcus*, *Staphylococcus*, and *Aerococcus* showed significant shifts in abundance. Statistical comparisons (Table S4) revealed that *Neisseria* was significantly more abundant in S1, while *Bacillus* and *Staphylococcus* showed increased abundance in S3. These genus-level shifts indicate potential functional changes in bacterial communities throughout fruit development.

#### 2.1.2. Fungal Community Composition and Structure

**Figure 3:**
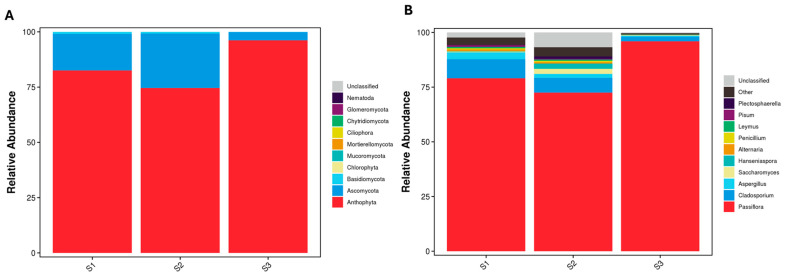
Relative abundance of fungal communities in the fruit juices of three developmental stages of *P. ligularis*. (**A**) Stacked bar chart displaying fungal phyla across S1, S2, and S3. (**B**) Stacked bar chart showing fungal genus-level composition. The analysis was based on ITS sequencing. S1, S2, and S3 represent the young fruit stage (0–45 days after fruit setting), coloration stage (46–59 days after fruit setting), and maturity stage (60–79 days after fruit setting), respectively.

The ITS sequencing analysis identified three dominant fungal phyla: Ascomycota (83.71%), Basidiomycota (15.68%), and Mucoromycota (0.59%), which altogether accounted for 99.9% of the total fungal community (Figure 3A). Ascomycota abundance increased in S2, while Basidiomycota declined over time. The abundance of Ascomycota and Basidiomycota was significantly higher in S1 than in S3, and the abundance of Mucoromycota was higher in S1 and S2 than in S3 (Table S5).

At the genus level, 206 fungal genera were detected (Figure 3B). *Passiflora* was the dominant fungal genus, increasing from S1 (79.03%) to S3 (95.94%), while *Cladosporium* and *Aspergillus* declined significantly. Statistical analysis (Table S6) confirmed significant changes in fungal genera, particularly *Aspergillus*, *Fusarium*, and *Acremonium*, across fruit developmental stages.

#### 2.1.3. Alpha Diversity Indices of the Endophytic Bacterial and Fungal Communities in the Developing *Passiflora ligularis* Fruit Juice

**Figure 4:**
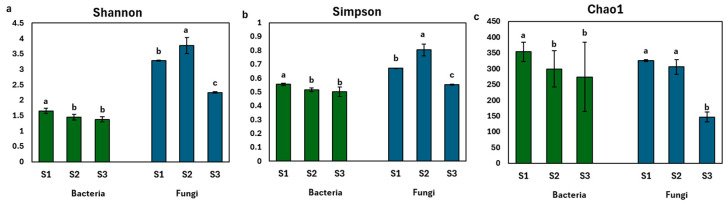
Alpha diversity indices, (**a**) Shannon, (**b**) Simpson, and (**c**) Chao1 of the endophytic bacterial and fungal communities in the developing *P. ligularis* fruit juice. S1, S2, and S3 represent the young fruit stage (0–45 days after fruit setting), coloration stage (46–59 days after fruit setting), and maturity stage (60–79 days after fruit setting) of *P. ligularis* fruit, respectively. Different letters in columns indicate significant differences (*p* < 0.05, *n* = 3).

Alpha diversity analysis (Shannon, Simpson, and Chao1 indices) showed a significant reduction in bacterial and fungal diversity from the young fruit stage (S1) to the maturity stage (S3), indicating microbial community shifts during fruit development (Figure 4). The complete alpha diversity dataset, including Observed_OTUs and Chao1 indices, is provided in Table S7.

#### 2.1.4. Beta Diversity Indices of the Endophytic Bacterial and Fungal Communities in the Developing *Passiflora ligularis* Fruit Juice

**Figure 5:**
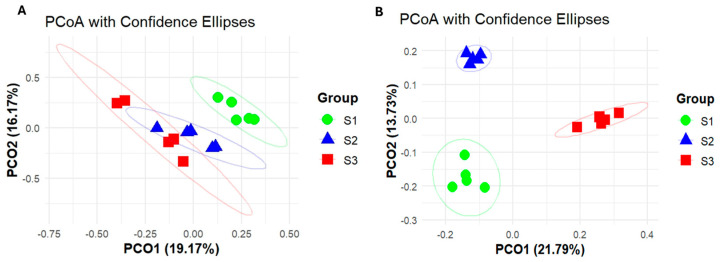
Principal coordinate analysis (PCoA) of the endophytic bacterial and fungal communities in the fruit juices of three developmental stages of *P. ligularis* fruit. (**A**) PCoA of the endophytic bacterial community. (**B**) PCoA of the endophytic fungal community. S1, S2, and S3 represent the young fruit stage (0–45 days after fruit setting), coloration stage (46–59 days after fruit setting), and maturity stage (60–79 days after fruit setting) of *P. ligularis* fruit, respectively.

On the basis of the first principal component (19.17% contribution), the endophytic bacterial communities of the S2 and S3 fruit juices were clustered in the same axis, separate from the endophytic bacterial communities of the S1 fruit juice (Figure 5A). The endophytic fungal communities in S1 and S2 were clustered separately from the endophytic fungal community in S3 according to the first principal coordinate (21.79% contribution) (Figure 5B). The endophytic fungal community in the S1 fruit juice was distinguished from that of the S2 fruit juice according to the second principal coordinate (13.73% contribution).

### 2.2. Volatile and Non-Volatile Flavor Metabolites in Developing Passiflora ligularis Fruit Juice

Using mass spectrometry, 87 volatile and 1002 non-volatile metabolites were identified across the three developmental stages of *P. ligularis* fruit juice. The molecular identities were assigned based on spectral similarity scores, retention indices, and, where possible, verification with authentic standards. To ensure high confidence in metabolite identifications, we primarily focused on compounds with high spectral similarity scores (>80%) while acknowledging other potentially important metabolites with lower confidence scores.

#### 2.2.1. Volatile Metabolites

A total of 87 volatile compounds were identified across the three developmental stages of *P. ligularis* fruit. These metabolites were classified into 28 distinct categories (Table S8). The relative proportion of these categories varied with the most abundant classes ranked as follows: saturated hydrocarbons > fatty alcohols > ethers > fatty alcohol esters > monoterpenoids = others > benzoic acid esters = fatty acids = organooxygen compounds = phenylpropanes = sesquiterpenoids > benzoic acids and derivatives = carbonyl compounds = lactones = unsaturated aliphatic hydrocarbons > alcohols and polyols = anisoles = benzene and substituted derivatives = benzoyl derivatives = benzyl alcohols = carbohydrates = carboxylic acid esters = fatty aldehydes = heptane oxide = ketones = lineolic acids = medium-chain aldehydes = oxanes = purine nucleosides. The data were subjected to a partial least squares-discriminant analysis (PLS-DA) (Figure S1), and the volatile flavor metabolites of S2 and S3 fruit juices were grouped separately from the volatile flavor metabolites of S1 fruit juices on the basis of the first principal component.

**Figure 6:**
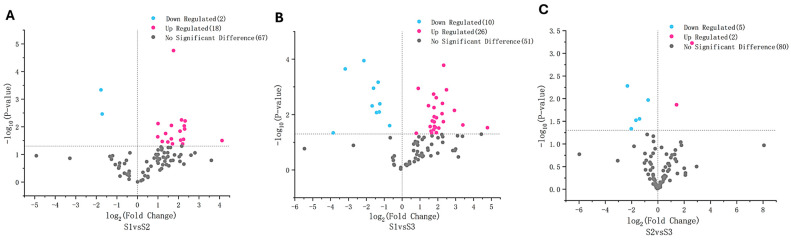
Volcano plots illustrate differentially accumulated volatile flavor metabolites across three developmental stages of *P. ligularis* fruit. (**A**) S1 vs. S2, (**B**) S1 vs. S3, and (**C**) S2 vs. S3. S1, S2, and S3 correspond to the young fruit stage (0–45 days after fruit setting), coloration stage (46–59 days after fruit setting), and maturity stage (60–79 days after fruit setting), respectively.

Among these, saturated hydrocarbons (14) and fatty alcohols (13) were the most abundant volatile metabolites (Table S8). Pairwise comparisons of the three developmental stages revealed significant differences in volatile flavor metabolite composition based on the criteria of variable importance in projection (VIP) > 1 and *p* < 0.05. The comparisons between S2 and S3 had the fewest metabolites with significant differences (7), whereas the comparison between S1 and S3 exhibited the highest number of differentially abundant volatile metabolites (30) (Figure 6; Table S9). Only three volatile metabolites exhibited substantial differences in the relative contents across developmental stages, with a fold-change greater than 10. These included 3-methylbenzaldehyde (benzoyl derivative), decanal (medium-chain aldehyde), and (*E*)-2-hexenyl benzoate (benzoic acid esters). The most pronounced change (27-fold) was observed for 3-methylbenzaldehyde in the comparison between S1 and S3 (Table S9).

Due to the complexity and variability of volatile metabolites, KEGG pathway enrichment analysis could not be performed. Only 3 of the 87 identified volatile compounds could be matched to the KEGG database, making enrichment analysis unfeasible. Unlike non-volatile metabolites, which have more well-characterized biosynthetic pathways, volatile compounds detected through headspace techniques undergo dynamic changes and are not comprehensively cataloged in KEGG. This limitation highlights the need for further database development to integrate volatile metabolite pathways.

#### 2.2.2. Non-Volatile Metabolites

**Figure 7:**
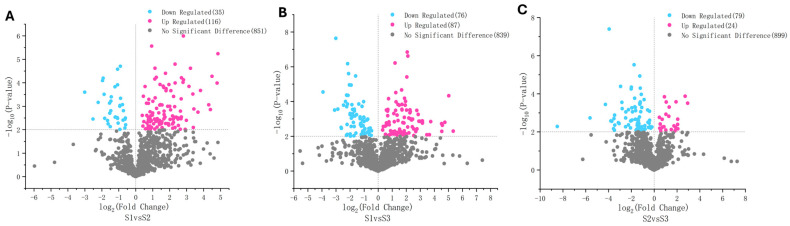
Volcano plots illustrate differentially accumulated non-volatile flavor metabolites across three developmental stages of *P. ligularis* fruit. (**A**) S1 vs. S2, (**B**) S1 vs. S3, and (**C**) S2 vs. S3. S1, S2, and S3 correspond to the young fruit stage (0–45 days after fruit setting), coloration stage (46–59 days after fruit setting), and maturity stage (60–79 days after fruit setting), respectively.

The non-volatile flavor metabolites in the developing *P. ligularis* fruit were analyzed using LC-MS/MS. A total of 1002 non-volatile metabolites were identified across the three developmental stages. These metabolites were classified into 13 superclasses (Figure 7; Table S10) with their relative proportions ranked as follows: organic acids and derivatives > lipids and lipid-like molecules > organoheterocyclic compounds > benzenoids > organic oxygen compounds > phenylpropanoids and polyketides > alkaloids and derivatives > organic nitrogen compounds > nucleosides, nucleotides, and analogues > organosulfur compounds > lignans, neolignans, and related compounds > hydrocarbons = mixed metal/non-metal compounds. The data were subjected to a PLS-DA, which clearly separated the three fruit juices in the PC1 × PC2 score plots (Figure S2). On the basis of the first coordinate, the non-volatile flavor metabolites in the S1 and S2 fruit juices were generally grouped separately from the non-volatile flavor metabolites in the S3 fruit juice.

Significant differences in non-volatile flavor metabolite composition were identified based on VIP > 1 and *p*-value < 0.01. A total of 140, 136, and 110 differentially accumulated non-volatile flavor metabolites were detected in the S1 vs. S2, S1 vs. S3, and S2 vs. S3 comparisons, respectively (Table S11). The predominant superclass with differentially accumulated metabolites was organic acids and derivatives in the S1 vs. S2 and S1 vs. S3 comparisons, while lipids and lipid-like molecules were the major differentially accumulated superclass in the S2 vs. S3 comparison.

Several non-volatile metabolites exhibited relative content differences exceeding 10-fold between developing stages (Figure 7). These included five benzenoids (tebuconazole, pargyline, bisoprolol, sufentanyl, and verapamil), three lipids and lipid-like molecules (talatisamine, baccatin, and 7*α*,27-dihydroxycholesterol), one organic acid and derivative (Ile-Tyr), one organic oxygen compound (*N*-acetylneuraminate), two organoheterocyclic compounds (hypoxanthine and methotrexate), and one phenylpropanoid and polyketide (aflatoxin) in the S1 vs. S2 comparison. Similarly, one alkaloid and derivative (hydrocodone), one benzenoid (tebuconazole), three organic oxygen compounds (*D*-xylulose, acetohexamide, and droperidol), one organoheterocyclic compound (hypoxanthine), and one phenylpropanoid and polyketide (aflatoxin) were identified in the S1 vs. S3 comparison. Additionally, one alkaloid and derivative (cabergoline), nine lipids and lipid-like molecules (LPC 18:1, cevadine, 2-linoleoylglycerol, solasodine, withaferin, cucurbitacin, baccatin, diacetoxyscirpenol, and (9*E*,11*E*)-octadecadienoic acid), one organic acid and derivative (Ile-Asp), and two organoheterocyclic compounds (olanzapine and pyraclostrobin) were identified in the S2 vs. S3 comparison. The extent of differential accumulation was notably lower in the S2 vs. S3 comparison than in the other two comparisons. The highest fold-change (360-fold) was observed for solasodine (lipid and lipid-like molecules) in S2 vs. S3 comparison (Table S11).

**Figure 8:**
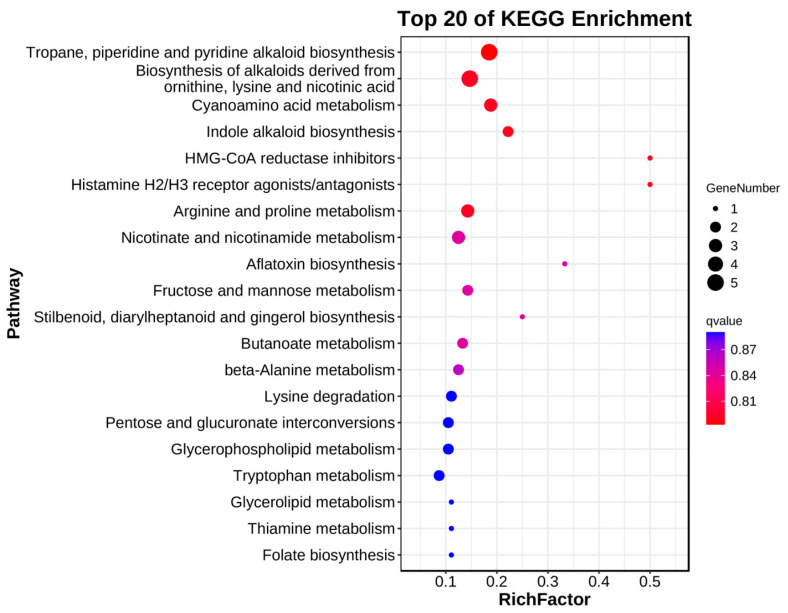
KEGG pathway enrichment analysis of differentially accumulated non-volatile metabolites in *Passiflora ligularis* fruit across three developmental stages. The y-axis represents the top 20 significantly enriched KEGG pathways, while the x-axis indicates the RichFactor (the ratio of the number of differentially accumulated metabolites annotated in a pathway to the total number of metabolites in that pathway). The size of the dots represents the number of metabolites mapped to each pathway, while the color represents the *q*-value (adjusted *p*-value), with red indicating more significant enrichment.

To further explore the metabolic pathways associated with non-volatile metabolites, KEGG pathway enrichment analysis was conducted. The analysis identified several significantly enriched pathways, providing insights into key metabolic processes associated with fruit development and flavor formation (Figure 8). The most significantly enriched pathways included arginine and proline metabolism, nicotinate and nicotinamide metabolism, glycerophospholipid metabolism, tryptophan metabolism, and beta-alanine metabolism. These pathways play crucial roles in amino acid metabolism, lipid biosynthesis, and secondary metabolite production, all of which contribute to the biochemical changes underlying fruit maturation and flavor development.

Additionally, pathways related to stilbenoid, diarylheptanoid, gingerol biosynthesis, and indole alkaloid biosynthesis were enriched, suggesting the involvement of specialized metabolite synthesis during the developmental stages of *P. ligularis* fruit. The results provide valuable insights into the biochemical processes shaping the fruit’s non-volatile flavor profile and emphasize the importance of metabolic regulation in fruit ripening.

### 2.3. Correlation Between Metabolites and Endophytic Microbiota

Understanding the interactions between metabolites and microbial communities is essential to elucidate the potential role of endophytic bacteria and fungi in shaping the metabolic profile of *P. ligularis* fruit. We analyzed the correlation between differentially accumulated volatile and non-volatile metabolites and dominant bacterial and fungal taxa at both the phylum and genus levels. These correlations provide insights into possible microbial influences on fruit metabolism and flavor development.

#### 2.3.1. Correlation Between Volatile Metabolites and Endophytic Microbiota

**Figure 9:**
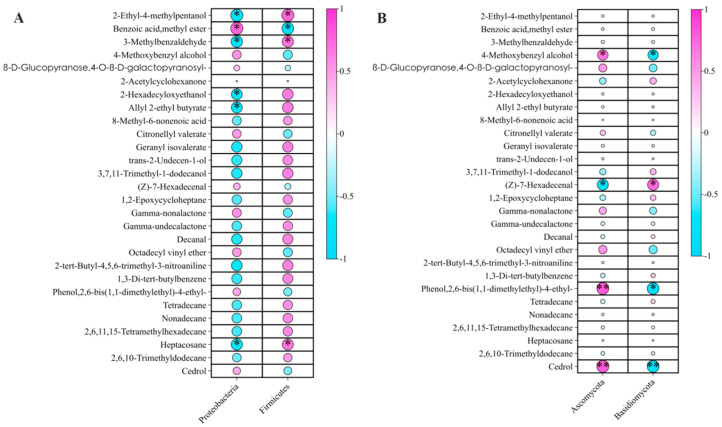
Correlations between 28 differentially abundant volatile flavor metabolites and the dominant endophytic microbiota with a relative abundance of ≥10% across three developmental stages of *P. ligularis* fruit. (**A**) Correlations with two dominant endophytic bacterial phyla. (**B**) Correlations with two dominant endophytic fungal phyla. * and ** indicate significant differences at *p* < 0.05 and *p* < 0.01, respectively.

Among the 87 identified volatile flavor metabolites, 28 of which exhibited significant variation across the three developmental stages (*p* < 0.01, q < 0.01), belonging to nine superclasses, including alcohols and polyols, benzoic acid esters, fatty alcohols, lactones, and phenylpropanes (Figure 9; Table S12). To investigate microbial influences on volatile metabolite accumulation, correlations were analyzed between these 28 differentially abundant volatiles and the dominant endophytic bacterial and fungal phyla (mean relative abundance ≥10%).

Among the bacterial phyla, Actinobacteria exhibited a positive correlation with benzoic acid methyl ester (*r* = 0.76*), a compound known for its aromatic properties (Figure 9A; Table S12). However, it showed negative correlations with 2-ethyl-4-methylpentanol (*r* = −0.76*), 3-methylbenzaldehyde (*r* = −0.71*), 2-hexadecyloxyethanol (*r* = −0.67*), allyl 2-ethyl butyrate (*r* = −0.67*), and heptacosane (*r* = −0.69*), suggesting the involvement of Actinobacteria in the degradation or transformation of volatile compounds. Firmicutes displayed significant positive correlations with 2-ethyl-4-methylpentanol (*r* = 0.75*), 3-methylbenzaldehyde (*r* = 0.69*), and heptacosane (*r* = 0.68*) but significantly negatively correlated with benzoic acid, methyl ester (*r* = −0.73*).

At the fungal phylum level, Ascomycota exhibited positive correlations with 4-methoxybenzyl alcohol (*r* = 0.70*), 2,6-bis(1,1-dimethylethyl)-4-ethylphenol (*r* = 0.80**), and cedrol (*r* = 0.81**) (Figure 9B; Table S13). In contrast, Ascomycota was significantly negatively correlated with (*Z*)-7-hexadecenal (*r* = −0.74*). Basidiomycota was negatively correlated with 4-methoxybenzyl alcohol (*r* = −0.68*), 2,6-bis(1,1-dimethylethyl)-4-ethylphenol (*r* = −0.79*), and cedrol (*r* = −0.80**) while showing a positive correlation with (*Z*)-7-hexadecenal (*r* = 0.74*). This suggests that Basidiomycota may contribute to the breakdown or transformation of specific volatile compounds as the fruit matures.

**Figure 10:**
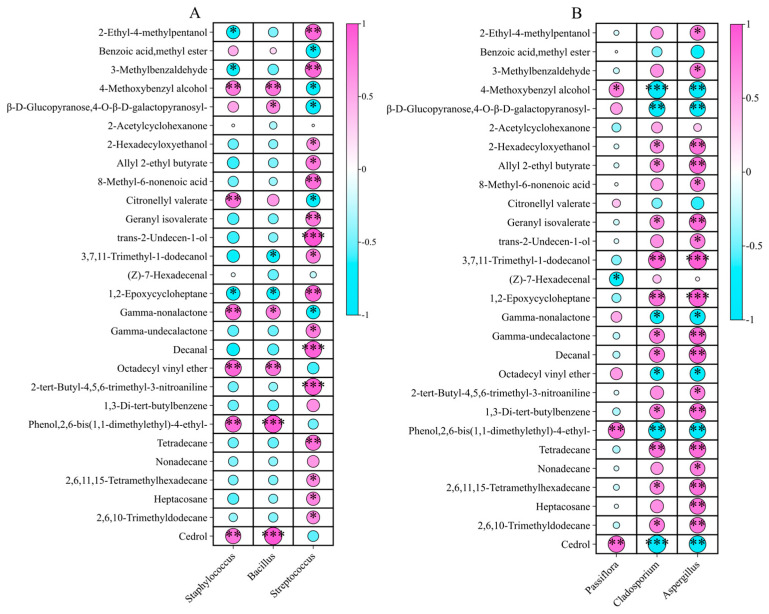
Correlations between 28 differentially abundant volatile flavor metabolites and the endophytic microbiota in the fruit juices of three developmental stages of *P. ligularis* fruit. (**A**) Correlations with three dominant bacterial genera. (**B**) Correlations with three dominant fungal genera. *, **, and *** indicate significant differences at *p* < 0.05, *p* < 0.01, and *p* < 0.001, respectively.

At the genus level, correlation was observed between the top three bacterial genera and volatile metabolites (Figure 10A; Table S14). *Staphylococcus* was positively associated with 4-methoxybenzyl alcohol (*r* = 0.80**), citronellyl valerate (*r* = 0.83**), *γ*-nonalactone (*r* = 0.86**), octadecyl vinyl ether (*r* = 0.83**), 2,6-bis(1,1-dimethylethyl)-4-ethylphenol (*r* = 0.88**), and cedrol (*r* = 0.83**). *Staphylococcus* showed negative correlations with 2-ethyl-4-methylpentanol (*r* = −0.71*), 3-methylbenzaldehyde (*r* = −0.67*), and 1,2-epoxycycloheptane (*r* = −0.73*).

*Bacillus* exhibited positive correlations with most of the volatile metabolites such as 2,6-bis(1,1-dimethylethyl)-4-ethylphenol (*r* = 0.97, *p* < 0.001), cedrol (*r* = 0.97***), and *γ*-nonalactone, indicating a potential role in the biosynthesis or stabilization of these aroma compounds. However, *Bacillus* was negatively correlated with 2-ethyl-4-methylpentanol, 3-methylbenzaldehyde, and 1,2-epoxycycloheptane, suggesting it may contribute to the degradation or modification of these volatile compounds during fruit development.

*Streptococcus* exhibited positive correlations with several key volatiles, particularly *trans*-2-undecen-1-ol, octadecyl vinyl ether, and citronellyl valerate, indicating its possible involvement in fruit aroma formation. In contrast, negative correlations of *Streptococcus* were observed for 2-hexadecyloxyethanol, tetradecane, and nonadecane, suggesting that it may play a role in modulating hydrocarbon-related volatiles.

Among fungi, *Passiflora* was negatively correlated with (*Z*)-7-hexadecenal (*r* = −0.70*) but positively correlated with 2,6-bis(1,1-dimethylethyl)-4-ethylphenol (*r* = 0.83**) and cedrol (*r* = 0.86**) (Figure 10B; Table S15). Similarly, *Cladosporium* and *Aspergillus* exhibited positive correlations with most of the volatile flavor metabolites. *Cladosporium* was negatively correlated with 4-methoxybenzyl alcohol (*r* = −0.92***) and cedrol (*r* = −0.93***). *Aspergillus* showed a highly significant positive correlation with 3,7,11-trimethyl-1-dodecanol (*r* = 0.93***) and 1,2-epoxycycloheptane (*r* = 0.91***).

#### 2.3.2. Correlation Between Non-Volatile Flavor Metabolites and Endophytic Microbiota

**Figure 11:**
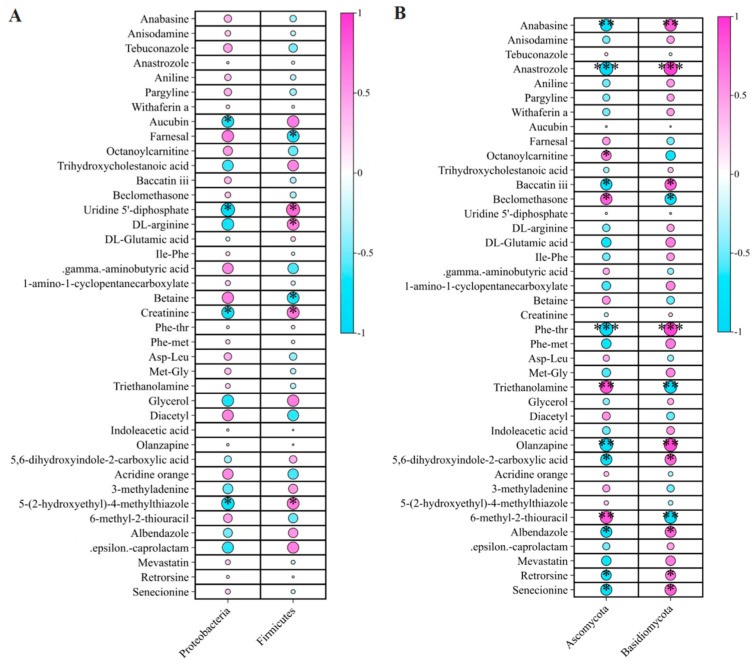
Correlations between 40 differentially abundant non-volatile flavor metabolites and the dominant endophytic microbiota (with a mean relative abundance of ≥10%) across three developmental stages of *P. ligularis* fruit. (**A**) Correlations with the two dominant endophytic bacterial phyla. (**B**) Correlations with the two dominant endophytic fungal phyla. *, **, and *** indicate significant differences at *p* < 0.05, *p* < 0.01, and *p* < 0.001, respectively.

To investigate microbial influences on non-volatile metabolite dynamics, we examined correlations between 40 differentially accumulated non-volatile metabolites and dominant bacterial and fungal taxa (Figure 11). These significantly different metabolites (ANOVA *p* < 0.01, *q* < 0.01) were classified into nine superclasses, including alkaloids and derivatives, benzenoids, lipids and lipid-like molecules, nucleosides, organic acids and derivatives, and organoheterocyclic compounds (Table S16).

Among bacterial phyla, Acidobacteria exhibited significantly negative correlations with aucubin (*r* = −0.67*), uridine 5′-diphosphate (*r* = −0.77*), creatinine (*r* = −0.70*), and 5-(2-hydroxyethyl)-4-methylthiazole (*r* = −0.72*) (Figure 11A; Table S16). *Firmicutes* displayed positive correlation with uridine 5′-diphosphate (*r* = 0.75*), *DL*-arginine (*r* = 0.67*), creatinine (*r* = 0.68*), and 5-(2-hydroxyethyl)-4-methylthiazole (*r* = 0.67*), but negatively correlated with farnesal (*r* = −0.67*) and betaine (*r* = −0.67*).

At the fungal phylum level, Ascomycota was positively correlated with triethanolamine (*r* = 0.86**), 6-methyl-2-thiouracil (*r* = 0.85**), octanoylcarnitine (*r* = 0.67*), and beclomethasone (*r* = 0.79*) (Figure 11B; Table S17). In contrast, Ascomycota was negatively correlated with nine metabolites, including anastrozole (*r* = −0.92***), olanzapine (*r* = −0.87**), and anabasine (*r* = −0.81**), and several alkaloids indicating its potential involvement in alkaloid metabolism and detoxification. In contrast, Basidiomycota exhibited strong positive correlations with alkaloids such as anastrozole (*r* = 0.92***), Phe-thr (*r* = 0.91***), olanzapine (*r* = 0.88**), anabasine (*r* = 0.81**), baccatin iii (*r* = 0.79*), senecionine (*r* = 0.78*), albendazole (*r* = 0.75*), and retrorsine (*r* = 0.69*), while negatively correlating with triethanolamine (*r* = −0.85**), 6-methyl-2-thiouracil (*r* = −0.84**), and beclomethasone (*r* = −0.78*).

**Figure 12:**
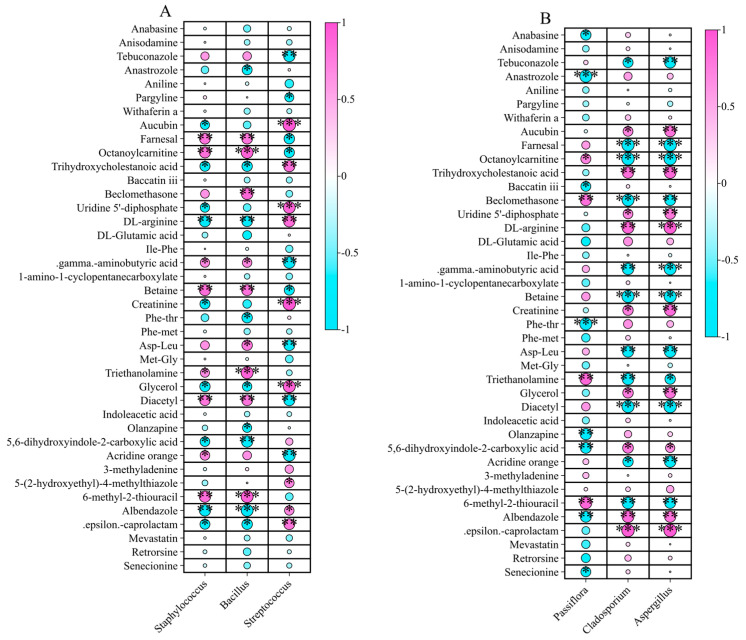
Correlations between 40 differentially abundant non-volatile flavor metabolites and the dominant endophytic microbiota (with a mean relative abundance of ≥10%) across three developmental stages of *P. ligularis* fruit. (**A**) Correlations with the three dominant bacterial genera. (**B**) Correlations with the three dominant fungal genera. *, **, and *** indicate significant differences at *p* < 0.05, *p* < 0.01, and *p* < 0.001, respectively.

At the bacterial genus level, *Staphylococcus* exhibited significant positive correlations with eight non-volatile metabolites and negative correlations with nine metabolites (Figure 12A; Table S18). *Bacillus* exhibited a strong positive correlation with octanoylcarnitine (*r* = 0.91***), triethanolamine (*r* = 0.92***), and 6-methyl-2-thiouracil and negative correlation with Albendazole (*r* = −0.91***). *Streptococcus* showed highly significant positive correlations with aucubin (*r* = 0.96***), uridine 5′-diphosphate (*r* = 0.90***), creatinine (*r* = 0.97***), and glycerol (*r* = 0.92***).

Among fungi, *Passiflora* exhibited negative correlations with anabasine (*r* = −0.77*), anastrozole (*r* = −0.92***), baccatin iii (*r* = −0.73*), Phe-thr (*r* = −0.92***), olanzapine (r = −0.85**), 5,6-dihydroxyindole-2-carboxylic acid (*r* = −0.81**), albendazole (*r* = −0.82**), and senecionine (*r* = −0.73*) (Figure 12B; Table S19). *Cladosporium* was negatively correlated with five metabolites, i.e., farnesal (*r* = −0.93***), octanoylcarnitine (*r* = −0.95***), beclomethasone (*r* = −0.90***), betaine (*r* = −0.92***), and diacetyl (*r* = −0.92***), but showed highly significant positive correlations with *ε*-caprolactam (*r* = 0.95***). *Aspergillus* displayed strong positive correlations with *DL*-arginine (*r* = 0.93***) and *ε*-caprolactam (*r* = 0.96***) and negative correlations with 11 non-volatile flavor metabolites.

## 3. Discussion

The fruit juice of plants belonging to the genus *Passiflora* is characterized by a balance of sweet and sour flavors, attributed to the presence of complex volatile components such as free volatile esters, sulfur-containing compounds, and norisoprene compounds. In this study, 87 volatile flavor metabolites were identified in the fruit juice of *P. ligularis* at different developmental stages. The number of metabolites detected in this study exceeds the number of volatiles reported for purple passion fruit (*P. edulis* Sims, 51 volatiles), yellow passion fruit (*P. edulis* Sims f. *flavicarpa* Deg., 42–44 volatiles), and banana passion fruit (*P. tarminiana*, 21 volatiles) [37,38], but remains lower than that of sweet passion fruit (*P. alata* Curtis, 103 volatiles) [39].

Among the identified volatile compounds, saturated hydrocarbons, fatty alcohols, and ethers were the dominant classes. Previous studies indicate that *P. edulis* fruit juice contains over 200 volatile compounds, with esters, terpenes, and sulfur-containing compounds playing critical roles in flavor development [40]. Other flavor metabolites, such as aldehydes, alcohols, ketones, ethers, phenols, and hydrocarbons, play a lesser role [40]. Esters, in particular, are key contributors to fruit aroma and accumulate during fruit ripening. At the same time, volatile esters accumulate gradually, and the aroma becomes more intense [41,42,43]. The lower presence of esters in *P. ligularis* compared to other *Passiflora* species may explain the milder aroma profile of its juice, as esters are recognized as primary determinants of fruit flavor intensity [44,45]. The variation in volatile metabolite composition among *Passiflora* species highlights the biochemical diversity within this genus and its influence on flavor characteristics.

Three distinct differentially abundant volatile flavor metabolites (3-methylbenzaldehyde, decanal, and (*E*)-2-hexenyl benzoate) were identified in the fruit juices of *P. ligularis* fruit. These compounds differ from the representative volatiles found in other *Passiflora* species, such as ethyl butyrate, ethyl caproate, and c-*β*-basil in yellow passion fruit (*P. edulis* Sims f. *flavicarpa* Deg.) [46]; ethyl butyrate, ethyl caproate, and beta-ionone in purple passion fruit (*P. edulis* Sims) [47]; (*Z*)-*β*-basil, *cis*-*β*-basil, hexyl butyrate, and hexyl caproate in banana passion fruit (*P. tarminiana*) [38,44]; methyl butyrate, ethyl butyrate, and ethyl caproate in sweet passion fruit (*P. alata* Curtis) [39]; methyl caproate, methyl 5-caproate, methyl acetate, and glyceryl acetate in pineapple passion fruit (*P. edulis* var. *violette*) [44]; hexyl butyrate and hexyl caproate in lemon passion fruit (*P. edulis* var. *panama gold*) [44]. This species-specific variation suggests that distinct volatile profiles contribute to the characteristic aroma of different *Passiflora* species, reinforcing the link between metabolic diversity and sensory attributes [38,39,44,45,46,47].

Regarding endophytic microbial communities, Proteobacteria and Firmicutes were the dominant bacterial phyla (relative abundance >10%) in the fruit juices of *P. ligularis* across all developmental stages. This finding is consistent with reports on endophytic bacterial communities in *Arabidopsis*, soybean, and rice, where these phyla dominate due to their role in plant–microbe interactions [48,49,50,51]. Previous studies showed that Proteobacteria in *Coffea arabica* is positively correlated with fruit size and caffeine content but negatively correlated with trigonelline accumulation [44,52]. Similarly, Firmicutes was the dominant endophyte in crops such as ginseng, sweet corn, tomato, watermelon, and bell pepper [53].

For endophytic fungi, Ascomycota and Basidiomycota were the predominant phyla (relative abundance >10%) in *P. ligularis* fruit, aligning with the previous studies reporting these as the dominant fungal group in phyllosphere environments [54,55,56]. Multiple factors, including plant genotype, developmental stage, and environmental conditions, can influence endophytic microbial diversity and abundance [50,57,58]. Studies on rice, maize, and eucalyptus confirm that the plant developmental stage plays a significant role in shaping the endophytic microbial community composition [59,60,61].

The microbial community structure in *P. ligularis* fruit juice varied significantly across developmental stages. Specifically, endophytic bacterial richness (Observed_OTUs and Chao1) and diversity (Shannon and Simpson) showed significant differences between the young fruit stage (S1) and the later stages (S2, S3). The fungal community richness (Observed_OTUs and Chao1) showed significant differences between the mature stage (S3) and other earlier stages (S1, S2), and the diversity (Shannon and Simpson) of the endophytic fungal community showed significant differences among the three developmental stages. The observed variations align with findings in *P. incarnata*, where bacterial community composition changed across developmental stages, likely in response to shifting physiological states [62]. These results reinforce the notion that *Passiflora* species regulate microbial associations throughout their growth cycles.

Plants require specific microbial interactions to meet physiological demands at different developmental stages. Plant metabolites are important agents of plant–microbial interactions, and their content depends on plant growth and development stage [63,64]. In the present study, Proteobacteria showed more negative correlations with volatile and non-volatile metabolites, whereas Firmicutes showed positive correlations with both metabolite types. Similarly, Ascomycota was positively correlated with volatile flavor metabolites, while Basidiomycota exhibited more negative correlations with volatile metabolites but positive correlations with non-volatile metabolites. This pattern suggests that microbial communities may actively influence fruit metabolite composition, potentially through enzymatic bio-transformations. Research on *Salvia miltiorrhiza* demonstrated that microbial communities contribute to the biosynthesis of secondary metabolites such as tanshinones, enhancing plant biomass [65]. Similarly, seed-associated bacterial and fungal microbial communities in *S. miltiorrhiza* have been linked to the accumulation of medicinal compounds [66]. Endophytic bacteria like *Bacillus*, *Pseudomonas*, and *Oligotrophomonas* are known to promote the accumulation of secondary metabolism in plants [67], supporting the idea that microbial interactions shape fruit metabolite profiles. Moreover, a study on *Arabidopsis thaliana* revealed that Proteobacteria in roots increased while Firmicutes decreased when coumarin synthesis genes were disrupted, highlighting the reciprocal influence of microbiota on metabolites [68]. The modulation of root exudate metabolites such as benzoxazinoids and triterpenoids impacts fungal and bacterial communities [52,69]. Overall, this study underscores the intricate relationships between microbial communities and fruit metabolite composition in *P. ligularis*. The observed variations in microbial abundance and diversity across developmental stages suggest dynamic interactions between host metabolism and microbial populations. Future studies should investigate the functional roles of key microbial taxa in influencing fruit flavor and nutritional composition, paving the way for microbiome-assisted breeding strategies to enhance fruit quality.

## 4. Materials and Methods

### 4.1. Sample Collection

**Figure 13:**
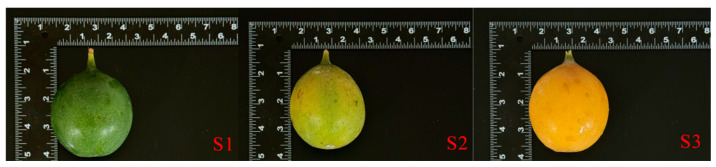
Three stages of development of *P. ligularis* fruit. (**S1**) Young fruit stage (45 days after fruit setting), (**S2**) coloration stage (60 days after fruit setting), (**S3**) maturity stage (80 days after fruit setting).

The *Passiflora ligularis* germplasm resource nursery of Tropical Crop College, Yunnan Agricultural University, is located in Puer City, Yunnan Province (101°08′~102°51′ E, 22°51′~23°59′ N), at an altitude of 1490 m. The area has an annual average temperature of 12.5 °C, with distinct dry and wet seasons. Experimental materials were selected from the three developmental stages of *P. ligularis* fruit from the field area at the College of Tropical Crops, Yunnan Agricultural University. The developmental stages included the young fruit stage (S1, 45 days after fruit set, 8 November 2024), the coloration stage (S2, 60 days after fruit set, 23 November 2024), and the maturity stage (S3, 90 days after fruit set, 13 December 2024) (Figure 13). For each developmental stage, 15 fruits were collected, with each group of five fruits constituting one biological replicate, resulting in three biological replicates per stage. The sterilization of the surface of the fresh *P. ligularis* fruit was to wipe the surface with 75% alcohol dipped in sterile paper. The fresh *P. ligularis* fruit juice was placed in a pre-weighed centrifuge tube and transported to the laboratory in an ice bath for further analysis of flavor components and endophytic microorganisms.

### 4.2. Sample Surface Disinfection and Total DNA Extraction

To ensure that only endophytic microorganisms were analyzed, surface disinfection of *P. ligularis* fruit samples was performed following a modified method by Sahu et al. [70]. The fruits were washed with sterile distilled water to remove soil particles and debris. They were then sequentially immersed in 70% ethanol for 1 min and 2% sodium hypochlorite (NaOCl) solution for 3 min and then rinsed three times with sterile distilled water. The final rinse water was plated onto tryptic soy agar (TSA) and potato dextrose agar (PDA) plates to confirm the absence of surface contaminants. Total genomic DNA of endophytic bacteria and fungi was extracted using the DNeasy Plant Mini Kit (Qiagen, Hilden, Germany) following the manufacturer’s protocol. The extracted DNA was quantified using a Nanodrop 2000 spectrophotometer (Thermo Fisher Scientific, Waltham, MA, USA), and its integrity was assessed via 1% agarose gel electrophoresis.

### 4.3. Sequencing and Analysis of Endophytic Microbial Amplicon in Passiflora ligularis Fruit Juice

The community composition of endophytic bacteria and fungi in fruit juice was determined by high throughput sequencing using the Illumina MiSeq method. PCR amplification of the 16S rRNA gene V3–V4 region for endophytic bacterial communities was performed using primers 341F (5′-CCTACGGGNGGCWGCA-3′) and 805R (5′-GACTACHVGGGTATCTAATCC-3′). For fungal communities, the ITS2 region was amplified using primers ITS1FI2 (5′-GTGARTCATCGAATCTTTG-3′) and ITS2 (5′-TCCTCCGCTTATTGATATGC-3′). PCR amplification was performed in a 20 μL reaction system containing 4 μL FastPfu Bufer (5×), 2 μL dNTPs (2.5 mM), 10 ng fruit juice endophytic microbial DNA, 0.4 μL forward and reverse primers (5 μM each), 0.2 μL bovine serum albumin (BSA), and 0.2 μL BSA, and 0.4 μL FastPfu Polymerase. The PCR conditions were: 95 °C for 3 min denaturation, followed by 27 cycles for bacteria and 35 cycles for fungi (95 °C 30 s, 55 °C 30 s, 72 °C 45 s), and a final extension at 72 °C for 10 min. Each sample was amplified in triplicate.

PCR products were purified with AxyPrep DNA gel extraction kit (Axygen Biosciences, Union City, CA, USA) and quantified using the QuantiFluor™-ST fluorescence quantification system. Amplicons were pooled in equimolar concentrations and sequenced on the Illumina MiSeq PE300 platform at Shanghai Biotree Biomedical Technology Co., LTD (Illumina Inc., San Diego, CA, USA). Raw sequence data were processed using QIME (version 1.17) for quality control and FLASH (version 1.2.7) for sequence assembly. The following criteria were applied: (1) Reads with a base quality score below 20 were trimmed; (2) reads shorter than 50 bp after quality filtering were discarded; (3) paired-end reads were merged using a minimum overlap length of 10 bp; (4) chimeric sequences were identified and removed using UCHIME.

Operational taxonomic units (OTUs) were clustered at 97% similarity using UPARSE (version 7.0.1090). Bacterial sequences were classified using the SILVA database, while fungal sequences were annotated based on the UNITE database (confidence threshold: 70%). To standardize sequencing depth, samples were rarefied to the minimum read count across all samples.

### 4.4. Liquid Chromatography–Tandem Mass Spectrometry Detection for Non-Volatile Flavor Components in Passiflora ligularis Fruit Juice

*P. ligularis* fruit juice (100 μL) was mixed with 400 μL of pre-cooled methanol/acetonitrile solution (1:1, *v*/*v*), vortexed, and incubated at −20 °C for 30 min. The mixture was centrifugated at 14,000× *g* at 4 °C for 20 min. The supernatant was vacuum-dried and reconstituted in 100 μL of acetonitrile/water (1:1, *v*/*v*) before LC-MS/MS analysis. Metabolites were separated using an Agilent 1290 Infinity LC ultra-high performance liquid chromatography (UHPLC) system (Agilent Corporatian, Santa Clara, CA, USA) with a HILIC column at 25 °C. Flow rate was 0.5 mL/min; Sample size was 2 μL; mobile phase composition A: water + 25 mM ammonium acetate + 25 mM ammonia water, B: acetonitrile. The gradient elution procedure is as follows: 0–0.5 min, 95% B; 0.5–7 min, B changes linearly from 95% to 65%; 7–8 min, B changes linearly from 65% to 40%; 8–9 min, B maintained at 40%; 9–9.1 min, B changes linearly from 40% to 95%; 9.1–12 min, B maintained at 95%. The samples were placed in a 4 °C automatic injector during the entire analysis process. In order to avoid the influence caused by the fluctuation of the instrument detection signal, the samples were analyzed continuously in random order.

An AB Triple TOF 6600 mass spectrometer was used to collect the primary and secondary spectra of the samples. The ESI Source conditions after HILIC chromatographic separation were as follows: Ion Source Gas1 (Gas1): 60; Ion Source Gas2 (Gas2): 60; Curtain gas (CUR): 30; source temperature: 600 °C; IonSapary Voltage Floating (ISVF) ±5500 V (positive and negative modes); TOF MS scan *m*/*z* range: 60–1000 Da; product ion scan *m*/*z* range: 25–1000 Da; TOF MS scan accumulation time 0.20 s/spectra; product ion scan accumulation time 0.05 s/spectra; secondary mass spectrometry was obtained using information-dependent acquisition (IDA) and high sensitivity mode, declustering potential (DP): ±60 V (positive and negative modes); collision energy: 35 ± 15 eV. IDA Settings were as follows: exclude isotopes within 4 Da; candidate ions to monitor per cycle: 10. The original data were converted by ProteoWizard (version 3.0.20365). Then XCMS program was used to perform peak alignment, retention time correction, and peak area extraction. Using XCMS (version 3.8.0) software, the parameters were set as follows: For peak picking, centWave *m*/*z* = 10 ppm; peakwidth = c (10, 60); prefilter = c (10, 100). For peak grouping, bw = 5, mzwid = 0.025, and minfrac = 0.5 were used.

The integrity of the data extracted by XCMS was checked first. Metabolites with more than 50% missing values in the group were removed. The empty KNN was filled, and the extreme values were deleted. Finally, the total peak area of the data was normalized to ensure a parallel comparison between samples and metabolites.

### 4.5. Gas Chromatography-Mass Spectrometry (GC-MS) for Volatile Flavor Compounds Analysis in Passiflora ligularis Fruit Juice

*P. ligularis* fruit juice (1 g) was placed in a headspace vial, mixed with saturated NaCl, and sealed with a TFE silicone cap (Agilent Corporatian, Santa Clara, CA, USA). Headspace solid-phase microextraction (HS-SPME) was conducted at 60 °C for 20 min using a 65 μm divinylbenzene/carboxyl/polydimethylsiloxane fiber (DVB/CAR/PDMS). After extraction, the volatile organic compounds were desorbed from the fiber coating at 250 °C for 5 min in the injection port of the GC instrument. Volatile organic compounds were identified and quantified using a Thermo Trace 1300 GC-MS system equipped with DB-wax column. Helium was used as the carrier gas and the linear speed was 1.0 mL/min. The injector temperature was maintained at 250 °C and the detector temperature at 280 °C. When the heating rate was 5/min, the oven temperature rose from 40 °C to 250 °C. All mass spectra were obtained in electronic impact (EI) mode with an ionization voltage of 70 eV. The quadrupole mass detector, ion source, and transmission line temperatures were set at 150 °C, 230 °C, and 280 °C, respectively. The mass spectrum was scanned in *m*/*z* 30~350 amu at intervals of 1 s. The isolated volatile compounds were identified by mass spectrometry compared with MWGC and linear retention index.

### 4.6. Statistics and Analysis

Partial least squares-discriminant analysis (PLS-DA) was conducted using R (version 4.3.1) software. In order to further understand the up- or down-regulated changes in the abundance of significantly changed metabolites (SCMs), the SCMs selected according to VIP value and *p*-value were combined with the fold change calculation in the comparison group to draw a volcano map. Pearson’s correlation analysis was performed to assess metabolic associations, and results were visualized using the R package “corrplot” (version 4.3.1).

## 5. Conclusions

This study analyzed volatile and non-volatile flavor metabolites in *Passiflora ligularis* fruit juices across three developmental stages, linking them to endophytic bacterial and fungal communities. A total of 87 volatile and 1002 non-volatile flavor metabolites were identified, with significant changes in non-volatile metabolites during fruit maturation. The endophytic communities, dominated by Firmicutes and Acidobacteria (bacteria) and Ascomycota and Basidiomycota (fungi), varied across stages. Key genera like *Staphylococcus*, *Bacillus*, and *Streptococcus* showed strong correlations with specific flavor compounds, suggesting their roles in aroma and metabolic processes. Such as a positive correlation of *Staphylococcus* with 4-methoxybenzyl alcohol, citronellyl valerate, and *γ*-nonalactone suggests its role in fruit aroma development. Similarly, *Bacillus* was positively correlated with 2,6-bis(1,1-dimethylethyl)-4-ethylphenol and cedrol but negatively correlated with 3-methylbenzaldehyde and 1,2-epoxycycloheptane, indicating its involvement in modifying volatile compounds. *Streptococcus* was positively correlated with *trans*-2-undecen-1-ol and octadecyl vinyl ether, which may influence fruit aroma composition. For non-volatile metabolites, *Bacillus* showed positive correlations with metabolites, indicating its role in lipid and nitrogen metabolism. *Streptococcus* exhibited positive correlations with non-volatile metabolites involved in nucleotide and amino acid metabolism. *Passiflora* showed a negative correlation with alkaloids, pointing to its potential role in alkaloid metabolism, while *Aspergillus* displayed strong positive correlations with amino acid metabolism. KEGG pathway analysis highlighted enrichment in lipid metabolism, amino acid biosynthesis, and secondary metabolite pathways. These findings reveal the interplay between flavor metabolites and endophytic microbiota, offering insights for improving fruit quality and postharvest management.

## Data Availability

Data are contained within the article and Supplementary Material.

## Supplementary Materials

The following supporting information can be downloaded at https://www.mdpi.com/article/10.3390/ijms26052151/s1.
