# Supplementary material for: Microbiome and Metabolome Illustrate the Correlations Between Endophytes and Flavor Metabolites in Passiflora ligularis Fruit Juice
Source: Int J Mol Sci. 2025 Feb 27;26(5):2151. doi: 10.3390/ijms26052151 (PMC11900049; doi:10.3390/ijms26052151)
Supplement: Supplementary file 1 [file ijms-26-02151-s001.zip › ijms-3458063-supplementary/Supplementary/Figure Supplementary.pdf]

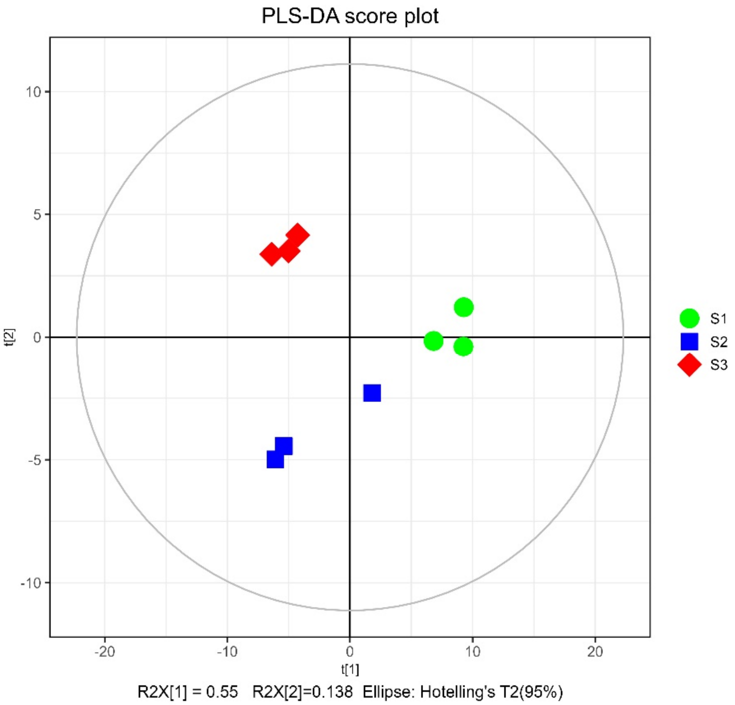

Figure S1. PLS-DA scores plot for volatile flavor metabolites in the fruit juices of three developmental stages of P. ligularis fruit.

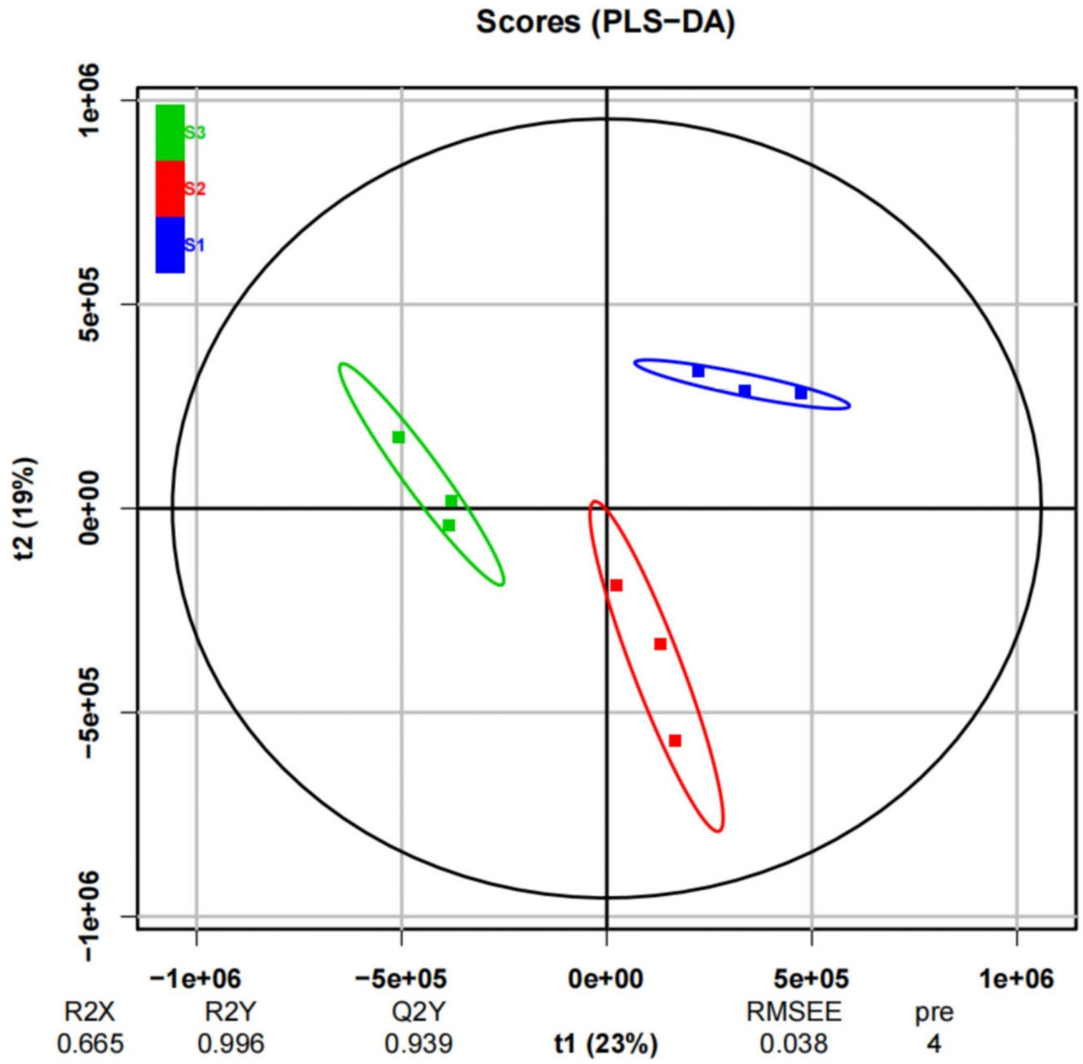

Figure S2. PLS-DA scores plot for non-volatile flavor metabolites in the fruit juices of three developmental stages of P. ligularis fruit.
