# Supplementary material for: Microbiome and Metabolome Illustrate the Correlations Between Endophytes and Flavor Metabolites in Passiflora ligularis Fruit Juice
Source: Int J Mol Sci. 2025 Feb 27;26(5):2151. doi: 10.3390/ijms26052151 (PMC11900049; doi:10.3390/ijms26052151)
Supplement: Supplementary file 1 [file ijms-26-02151-s001.zip › ijms-3458063-supplementary/Supplementary/Table Supplementary.pdf]

Table S1. Statistical tables of valid data of bacterial OTUs in the fruit juices of three developmental stages of *P. ligularis* fruit.

Sample	Tags number	Total length	Max length	Min length	N50	N90
S1-1	116474	47313421	431	384	429	429
S1-2	107400	43618882	430	211	429	429
S1-3	114626	46566048	433	233	429	429
S2-1	106583	43295314	430	249	429	429
S2-2	119278	48437622	430	249	429	429
S2-3	113727	46204665	430	310	429	429
S3-1	108138	43943585	431	249	429	429
S3-2	110053	44722410	430	246	430	430
S3-3	107777	43786703	430	249	430	430

S1, S2, and S3 represents young fruit stage (45 days after fruit setting), coloration stage (60 days after fruit setting), and maturity stage (80 days after fruit setting) of *P. ligularis* fruit, respectively.

Table S2. Statistical tables of valid data of fungal OTUs in the fruit juices of three developmental stages of *P. ligularis* fruit.

Sample	Tags number	Total length	Max length	Min length	N50	N90
S1-1	123768	43414727	435	211	419	419
S1-2	124938	43837800	435	235	419	419
S1-3	126745	44399235	435	208	419	419
S2-1	114046	39880904	439	211	419	419
S2-2	122259	41651021	435	209	419	419
S2-3	116497	40557036	435	226	419	419
S3-1	123152	44072064	435	218	377	377
S3-2	120691	43220447	435	215	377	377
S3-3	125388	44809399	435	211	377	377

S1, S2, and S3 represent the young fruit stage (45 days after fruit setting), coloration stage (60 days after fruit setting), and maturity stage (80 days after fruit setting) of *P. ligularis* fruit, respectively.

Table S3. The phyla of bacterial communities in terms of relative abundance in pairwise comparisons in the fruit juices of three developmental stages of *P. ligularis* fruit.

Bacterial phylum	P value	Relative abundance comparison between fruit juice sample groups
Proteobacteria	0.05	S1 (56.44) vs S2 (62.19)
Firmicutes	0.05	S1 (42.33) vs S2 (37.01)
Bacteroidota	0.05	S1 (0.19) vs S2 (0.10)

S1, S2, and S3 represent the young fruit stage (0-45 days after fruit setting), coloration stage (46-59 days after fruit setting), and maturity stage (60-79 days after fruit setting) of *P. ligularis* fruit, respectively.

Table S4 The genera of bacterial communities in terms of relative abundance in pairwise comparisons in the fruit juices of three developmental stages of *P. ligularis fruit.*

**Bacterial genus**	**P value**	**Relative abundance comparison between fruit juice sample groups**
Neisseria	0.05	S1 (0.11) vs S2 (0.01)
		S1 (0.11) vs S3 (0.001)
Bacillus	0.05	S1 (0.02) vs S3 (0.32)
		S2 (0.04) vs S3 (0.32)
Streptococcus	0.05	S2 (0.01) vs S3 (0.005)
Aerococcus	0.05	S2 (0.05) vs S3 (0.01)
Staphylococcus	0.05	S1 (0.05) vs S3 (0.39)

S1, S2, and S3 represent young fruit stage (0-45 days after fruit setting), coloration stage (46-59 days after fruit setting), and maturity stage (60-79 days after fruit setting) of *P. ligularis* fruit, respectively.

Table S5. The phyla of fungal communities in terms of relative abundance in pairwise comparisons in the fruit juices of three developmental stages of *P. ligularis* fruit.

**Fungal phylum**	**P value**	**Relative abundance comparison between fruit juice sample groups**
Ascomycota	0.05	S1 (82.56) vs S3 (96.15)
Basidiomycota	0.05	S1 (16.54) vs S3 (3.74)
Mucoromycota	0.05	S1 (0.88) vs S3 (0.10)<br>S2 (0.76) vs S3 (0.10)

S1, S2, and S3 represent the young fruit stage (0-45 days after fruit setting), coloration stage (46-59 days after fruit setting), and maturity stage (60-79 days after fruit setting) of *P. ligularis* fruit, respectively.

Table S6 The genera of fungal communities in terms of relative abundance in pairwise comparisons in the fruit juices of three developmental stages of *P. ligularis* fruit.

Fungal genus	P value	Relative abundance comparison between fruit juice sample groups
Aspergillus	0.05	S1 (3.14) vs S2 (1.84)
		S1 (3.1) vs S3 (0.58)
		S2 (1.84) vs S3 (0.58)
Sarocladium	0.05	S1 (0.05) vs S2 (0.60)
		S2 (0.60) vs S3 (0.04)
Fusarium	0.05	S1 (0.03) vs S2 (0.12)
		S2 (0.12) vs S3 (0.05)
Acremonium	0.05	S1 (0.03) vs S2 (0.13)
		S1 (0.03) vs S3 (0.01)
		S2 (0.13) vs S3 (0.01)
Zygophiala	0.05	S1 (0.001) vs S2 (0.09)
		S2 (0.09) vs S3 (0.005)
Phaeosphaeria	0.05	S1 (0) vs S2 (0.08)
		S2 (0.08) vs S3 (0.008)
Penicillium	0.05	S1 (0.55) vs S3 (0.10)
		S2 (0.57) vs S3 (0.10)
Cladosporium	0.05	S1 (8.71) vs S3 (2.00)
		S2 (6.79) vs S3 (2.00)
Malassezia	0.05	S1 (0.46) vs S3 (0.06)
		S2 (0.32) vs S3 (0.06)
Passiflora	0.05	S1 (79.03) vs S3 (95.94)
Alternaria	0.05	S1 (0.78) vs S3 (0.03)
Leymus	0.05	S1 (0.61) vs S3 (0.04)
Pisum	0.05	S1 (0.78) vs S3 (0.03)
Glycine	0.05	S1 (0.58) vs S3 (0.04)
Xeromyces	0.05	S1 (0.41) vs S3 (0.01)
Claviceps	0.05	S1 (0.33) vs S3 (0.01)
Candida	0.05	S1 (0.21) vs S3 (0.03)
Brassica	0.05	S1 (0.20) vs S3 (0.01)
Epicoccum	0.05	S1 (0.14) vs S3 (0.01)
Vishniacozyma	0.05	S1 (0.07) vs S3 (0.003)
Sporobolomyces	0.05	S1 (0.04) vs S3 (0.0005)
Gibellulopsis	0.05	S2 (0.18) vs S3 (0.05)

S1, S2, and S3 represent young fruit stage (0-45 days after fruit setting), coloration stage (46-59 days after fruit setting), and maturity stage (60-79 days after fruit setting) of *P. ligularis* fruit, respectively.

Table S7. Alpha diversity indices of the endophytic bacterial communities in the developing *P. ligularis* fruit juice.

Samples	Observed_OTUs	Shannon	Simpson	Chao1	Goods_coverage
S1	353.67±25.00a	1.65±0.07a	0.55±0.01a	353.67±25.00a	1.00±0.00a
S2	299.00±46.93b	1.45±0.07b	0.52±0.01b	299.00±46.93b	1.00±0.00a
S3	274.00±90.07b	1.37±0.07b	0.50±0.03b	274.00±90.07b	1.00±0.00a
